# Intranasal administration of dauricine loaded on graphene oxide: multi-target therapy for Alzheimer's disease

**DOI:** 10.1080/10717544.2021.1895909

**Published:** 2021-03-17

**Authors:** Kaixuan Wang, Lingfeng Wang, Ling Chen, Chiwei Peng, Beijiao Luo, Jingxin Mo, Wei Chen

**Affiliations:** a School of Pharmacy, Guilin Medical University, Guilin, China; b School of Pharmacy, Zhejiang Chinese Medical University, Zhejiang, China; c Department of Pharmacy, Affiliated Hospital of Guilin Medical University, Guilin, China

**Keywords:** Alzheimer’s disease, beta-amyloid protein, oxidative stress, neuroprotection, apoptosis, cognition

## Abstract

Alzheimer's disease (AD) is a degenerative disease of the central nervous system characterized by progressive cognitive and memory-related impairment. However, current therapeutic treatments have not proved sufficiently effective, mainly due to the complicated pathogenesis of the disease. In this study, a nano-formulation of graphene oxide (GO) loaded with dauricine (Dau) was investigated in terms of the combined anti-inflammatory and anti-oxidative stress effects of Dau and the inhibition of misfolding and aggregation of the amyloid-β (Aβ) protein by GO. Both *in vivo* and *in vitro* models were induced using Aβ_1-42_, and the formulation was administered nasally in mice. The results showed that GO loaded with Dau greatly reduced oxidative stress through increasing superoxide dismutase levels and decreasing reactive oxygen species and malondialdehyde levels *in vitro*; it also alleviated the cognitive memory deficits and brain glial cell activation in mice with Aβ_1-42_-induced AD. This proved that GO loaded with Dau could protect against Aβ_1-42_-induced oxidative damage and apoptosis in both *in vitro* and *in vivo* AD models; therefore, GO loaded with Dau has the potential to be an effective and agent for the rapid treatment of AD.

## 1. Introduction

Alzheimer's disease (AD) is the most common form of dementia in the elderly. It is characterized by progressive development after an insidious onset, and no cure has been developed as yet. Thus, AD remains an enormous financial and social burden for patients and their families due to the terrible and devastating loss of cognition and decline in daily behavior it causes (Uzun et al., 2011; Fransquet et al., 2018). Moreover, the pathogenesis of AD is still unclear, and the simultaneous effects of a variety of causes are believed to be responsible for the onset of the disease. According to the most influential hypothesis of AD pathogenesis, the misfolding of the amyloid-β (Aβ) peptide to form toxic oligomers and fibrils in brain tissue is considered as a key factor in AD. The aggregation of neurotoxic Aβ leads to increased oxidative stress and inflammation in the brain, promoting neuronal apoptosis (Butterfield et al., 2013). This eventually results in chronic and irreversible brain tissue damage and multiple neurological impairments, resulting in significant memory deficits and impaired judgment (Butterfield & Boyd-Kimball, 2018; Ziegler-Waldkirch et al., 2018). Therefore, there is an urgent need to develop novel agents for the treatment of AD.

Dauricine (Dau) is a dibenzyl tetrahydroisoquinoline alkaloid extracted and isolated from the rhizome of *Menispermum dauricum*. Several studies have shown that Dau exhibited anti-proliferative activity against several different types of malignant cell (Zhang et al., 2018, 2019; Zhou et al., 2019). Dau can regulate the expression of Bcl-2 family proteins through the mitochondrial pathway, inhibit cell apoptosis, and have a protective effect against brain damage (Li & Gong, 2007). Studies have also shown that Dau down-regulated caspase-3, alleviated endoplasmic reticulum stress, and had protective effects in a nematode model of AD by stimulating the IRE-1/XBP-1 signaling pathway (Pu et al., 2018); it also had a strong antioxidant effect against acute oxidative damage (Wang et al., 2020). However, the short half-life, rapid metabolism, and certain cytotoxic effects of Dau (Wei et al., 2015; Liu & Liu, 2016) greatly limit its applicability for the treatment of AD.

Recently, multifunctional drug-loaded nanosystems have emerged as an effective treatment strategy to treat complex and refractory diseases (Yoon et al., 2015; Jansook et al., 2018; Lv et al., 2018). As the processing product of graphene, graphene oxide (GO) has several advantages over in terms of higher specific surface area, better water solubility and biocompatibility. Polymers and drugs can be linked to GO particles by covalent or non-covalent binding, resulting in high drug loading (Parviz & Strano, 2018). Moreover, low concentrations of GO could degrade the Aβ protein and reduce Aβ-induced cell apoptosis (Yang et al., 2015). Thus, drug delivery systems based on GO have the potential to be used as multi-targeted and more efficient therapeutic strategies for AD.

However, in the clinical setting, the most difficult problem in the treatment of AD is the unique blood–brain and blood–cerebrospinal fluid barrier of the central nervous system, which intercept toxic and macromolecular substances, but also block therapeutic drugs. This is also the main reason for the poor therapeutic efficacy and peripheral adverse reactions of AD drugs (Henrich-Noack et al., 2019; Langen et al., 2019). Therefore, intranasal administration, which enables noninvasive delivery from the nose to the brain, was used in animal experiments in this study – the drug is introduced from the nose into the cerebrospinal fluid, and then into the extracellular fluid of brain tissue after passing through the blood–brain barrier. The success of several nasal drugs on the market is indicative of the potential advantages over traditional administration routes (Kamei et al., 2018; Li et al., 2019).

In the present study, we investigated GO nanoparticles loaded with Dau (hereafter referred to as GO@Dau) as a novel therapeutic drug for AD and compared its *in vivo* and *in vitro* neuroprotective effects on Aβ_1-42_-induced AD with those of GO or Dau alone.

## 2. Materials and methods

### 2.1. Chemicals and reagents

Dau (purity > 98%) was procured from Chengdu Must Bio-Technology Co., Ltd. (Chengdu, China). GO dispersion was purchased from Jiangsu XFNANO Materials Tech. Co., Ltd. (Nanjing, China). Aβ_1-42_ was obtained from Jill Biochemical Co., Ltd. (Shanghai, China). Except for the CCK-8 assay kit (Dojindo, Kumamoto, Japan), all the other kits used were ordered from Beyotime (Shanghai, China). Monoclonal antibodies against α-tubulin, Bcl-2-associated X protein (Bax), Bcl-2, caspase-3, Kelch-like ECH-associated protein 1 (Keap1), nuclear factor erythroid 2-related factor 2 (Nrf2), brain-derived neurotrophic factor (BDNF), glial fibrillary acidic protein, and Iba-1 and horseradish peroxidase (HRP)-conjugated anti-rabbit/mouse IgG secondary antibody were purchased from Abcam (Cambridge, UK). All chemicals used were of high purity analytical grade.

### 2.2. Cell culture and preparation of aggregated Aβ_1-42_

Human SH-SY5Y cells were routinely cultured in Dulbecco’s modified Eagle medium containing 10% fetal bovine serum and 1% 100 U/mL penicillin and streptomycin in an incubator in 5% CO_2_ at 37 °C.

Aggregated Aβ_1-42_ was prepared as previously described (Ye et al., 2015). Briefly, Aβ_1-42_ was dissolved in hexafluoroisopropanol to a concentration of 2 mg/mL. The resulting solution was transferred into smaller tubes and evaporated in a biochemical fume hood, and the peptide membrane obtained was stored at −80 °C until further use. For the *in vitro* experiments, the Aβ_1-42_ peptide membrane was incubated at 37 °C for two days before use.

For animal experiments, Aβ_1-42_ was dissolved in 0.9% saline at a final concentration of 1 µg/µL, and incubated for seven days at 37 °C for aggregation (Xie et al., 2018).

### 2.3. Animals and drug administration

All experimental procedures using mice received specific approval from the Institutional Animal Ethics Committee (IAEC) of Guilin Medical University (protocol no.: GLMC-201905013), and all efforts were made to minimize animal suffering. Male C57BL/6 mice (aged 2–3 months, weight 20–28 g) were selected for *in vivo* experiments. Mice were housed at 22 ± 2 °C on a 12 h light/dark cycle with free access to water and standard rodent diet.

### 2.4. Synthesis and characterization of GO and GO@Dau

#### 2.4.1. Preparation of GO@Dau

First, in order to ensure the uniformity of GO particle size, Tween 80 was added to a final concentration of 1% in the GO dispersion, and then ultrasonic disrupted for 30 min, after which the solution was filtered through a 0.45 μm membrane filter and stored at 4 °C until further use. One milliliter of 0.5 mg/mL Dau was added to 25 mL of this GO dispersion and stirred at 25 °C for 24 h. Thereafter, the solution was filtered through a 0.45 μm membrane filter, centrifuged at 13,000 rpm and 4 °C for 1 h, and freeze-dried to obtain GO@Dau as the precipitate.

#### 2.4.2. Particle size and zeta potential

The particle size and zeta potential of GO and GO@Dau were measured using light scattering (NanoBrook 90Plus PALS; Brookhaven, NY).

#### 2.4.3. Transmission electron microscopy (TEM)

The percentage of drug loading was determined by stirring 5 mg GO@Dau in 100 mL phosphate-buffered saline (PBS) at pH 7.4 for 48 h. The obtained suspension was centrifuged and the Dau content was determined at 281 nm using high-performance liquid chromatography (HPLC) (LC-2030, Shimadzu, Kyoto, Japan). Drug loading percentage and encapsulation efficiency percentage were calculated using the following formulae:

$$\text{Percent drug loading }=\;(W_{\text{total dau}}-W_{\text{free dau}})/W_{\text{GO@Dau}}\times \text{ 1}00\%$$

$$\text{Percent encapsulation efficiency }=\;(W_{\text{total dau}}-W_{\text{free dau}})/W_{\text{total dau}}\times \text{ 1}00\%$$

### 2.5. Cell viability assay

The CCK-8 assay was used to determine cell viability as previously described (Cheng et al., 2018), Briefly, undifferentiated SH-SY5Y cells were seeded in 96-well plates (6000 cells/well) and allowed to adhere for 24 h at 37 °C in 5% CO_2_. Thereafter, the culture medium was removed and the cells were treated with the experimental drug medium. After appropriate incubation, the drug medium was removed and 10 μL of CCK-8 solution was added and incubated for 1–2 h at 37 °C. Absorbance was measured at 450 nm using a microplate reader (Infinite 200 Pro; Tecan, Grödig, Austria). The cell viability was calculated based on the absorbance ratio with reference to that in the control group.

### 2.6. Intracellular ROS level assay

The fluorescent probe 2′,7′-dichlorofluorescin diacetate (DCFH-DA) was used to measure intracellular ROS levels. Briefly, SH-SY5Y cells were seeded in six-well plates (8 × 10^4^ cells/well) and cultured for 24 h at 37 °C in 5% CO_2_. Culture medium was then removed and the following drugs were added: 0.1% DMSO (control group), 30 μM Aβ_1-42_ (model group), 30 μM Aβ_1-42_+2 µM Dau (Dau group), 30 μM Aβ_1-42_+1 µM Dau (OD group), 30 μM Aβ_1-42_+23 mg/mL GO (GO group), and 30 μM Aβ_1-42_+25 mg/mL GO@Dau (GO@Dau group, equivalent to 2 µM Dau). Here, we chose two concentrations of Dau – one was the optimal therapeutic concentration (in the OD group) and the other was the amount contained in GO@Dau (in the Dau group). After 48 h, the cells were incubated with DCFH-DA at a final concentration of 5 μM at 37 °C for 30 min and then washed with culture medium without serum three times. Fluorescent intensities at 488 nm/525 nm (excitation/emission) wavelength were determined and images were obtained using a fluorescence microscope (Olympus, Tokyo, Japan). The final data are represented in terms of the ratios of the fluorescence intensities with reference to the control group as quantified using ImageJ (1.49 V, NIH, Bethesda, MD).

### 2.7. Superoxide dismutase (SOD) and malondialdehyde (MDA) levels assay

Lipid peroxidation in SH-SY5Y cells was determined based on the levels of SOD and MDA using commercial kits. The SH-SY5Y cell drug treatment groups are as described in Section 2.6. Protein concentrations were measured using a BCA Assay kit. After treatment, the cells were collected and then processed according to the manufacturer's instructions for SOD level analysis.

Drug treated cells were washed with PBS, lysed in radioimmunoprecipitation assay (RIPA) buffer, and centrifuged at 12,000×*g* for 30 min at 4 °C. The supernatant was collected to analyze the MDA content.

### 2.8. Apoptosis assay

Apoptosis and necrosis were assessed using a one-step terminal deoxynucleotidyl transferase dUTP nick end labeling (TUNEL) apoptosis assay kit. The drug treatment groups were the same as described in Section 2.6, and samples were processed according to the manufacturer’s instructions. Fluorescent intensities at 550 nm/570 nm (excitation/emission) wavelength were determined and images were obtained using a fluorescence microscope. The final data are represented in terms of the ratios of the fluorescence intensities with reference to the control group as quantified using ImageJ.

### 2.9. Establishment of AD model and administration

The AD model was established by subjecting mice to an intracerebroventricular injection of aggregated Aβ_1-42_ (1 μg/μL) using a microinjector. Mice were anesthetized by an intraperitoneal injection of pentobarbital (0.4 g/kg body weight) and mounted on a stereotaxic frame (Stoelting, Wood Dale, IL). An incision was made in the skull along the cranial midline sagittal line to expose bregma completely. The location of the injection site was verified according to The Mouse Brain in Stereotaxic Coordinates, second edition: 2 mm posterior to the bregma, 1.5 mm from the biparietal suture, and 2 mm under the skull. Five microliters saline or aggregated Aβ_1-42_ (1 μg/μL) was injected into the brain using a microinjector over 5 min. After injection, the needle was left in place for another 5 min, and then pulled out slowly. The mice were given one day to recover, after which they were intranasally administrated with the appropriate drugs every other day.

**Figure 1.**
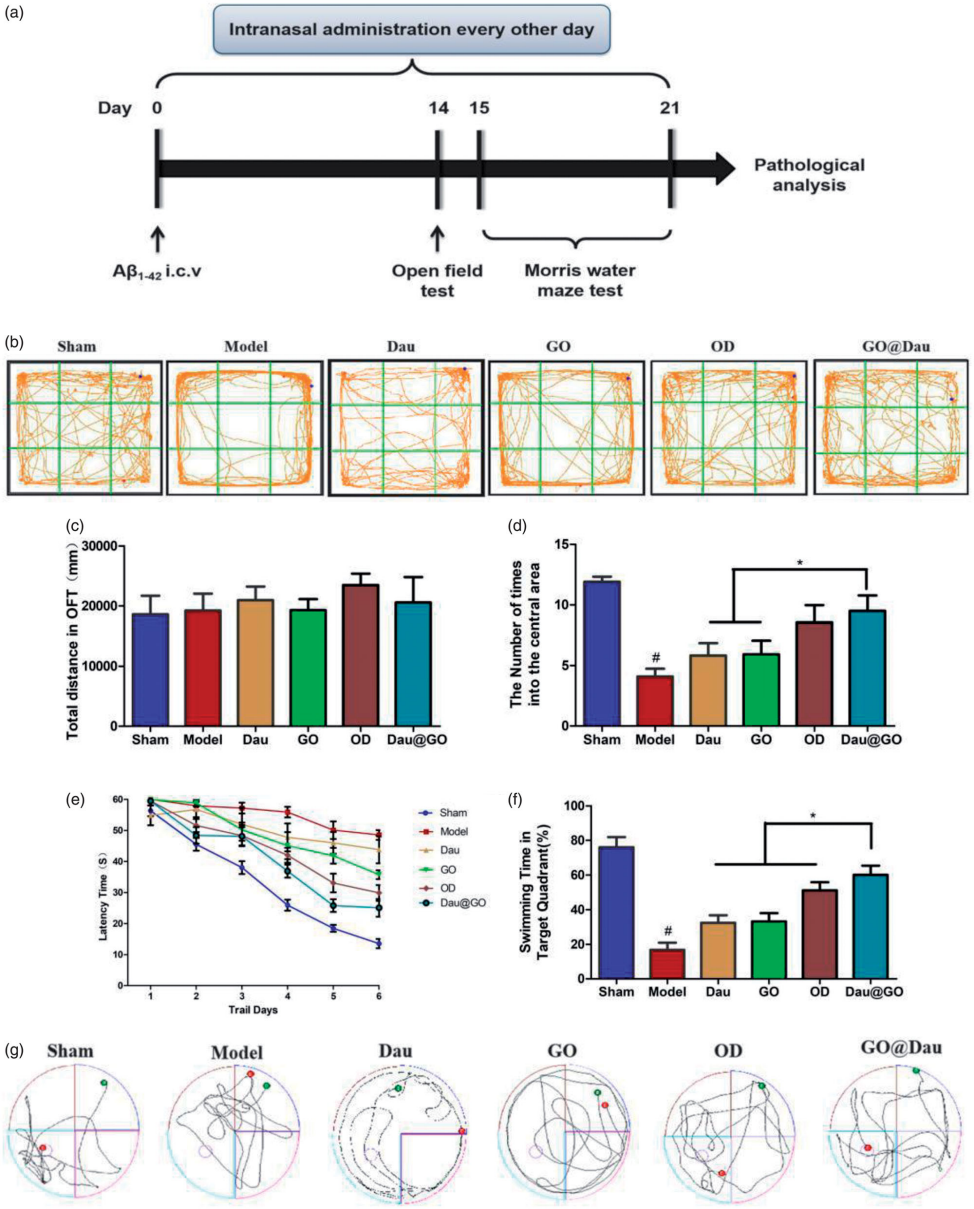
Effect of GO@Dau on anxiety and cognitive deficits in mice treated with aggregated Aβ_1-42_. (a) The schedule of animal experiment. (b) Representative traces of mouse movement during an OFT. (c) Total distance performed in OFT. (d) Number of times to cross the central area of the OFT. Time latency (e), the percentage of time spent in the target quadrant (f) and swimming track at the last trail day (g) of the MWM test (*n* = 10, *p < .05).

A total of 120 mice were used in this study. Twenty mice in the sham operation group were injected with 5 μL 0.9% saline into the lateral ventricle. The remaining 100 mice were randomly divided into five groups after AD surgery and treated as follows: the control group was subjected to AD surgery and treated with 0.9% saline; the Dau group was subjected to AD surgery, then treated with 0.03 g/kg Dau; the OD group (with the optimal concentration of Dau) was subjected to AD surgery and treated with 0.015 g/kg Dau; the GO group was treated with 0.40 g/kg GO after AD surgery; and the GO@Dau group was treated with 0.43 g/kg GO@Dau (equivalent to 0.03 g/kg Dau) after AD surgery. Behavioral experiments were performed using the open field test (OFT) and the Morris water maze (MWM) to evaluate memory and learning capacity from days 14 to 21. Finally, the mice were euthanized by intraperitoneal injection of pentobarbital (150 mg/kg), and brains were collected for pathological analyses and protein assessment. The schedule of animal experiments is shown in Figure 1.

### 2.10. Behavioral studies

We finally selected mice that survived after brain surgery and drug administration; we also screened out mice that could not crawl and swim. Behavioral study data were collected by two trained researchers blinded to the group allocations.

#### 2.10.1. OFT

The OFT was performed on the first day after the end of administration. Each mouse was placed in the open field box, and then activity was recorded using a camera for 10 min. The total distance traveled and the time spent in the central region were measured to evaluate the motor functioning and anxiety-like behavior of mice.

#### 2.10.2. MWM test

The spatial learning and memory abilities of the mice were assessed using the MWM test. All MWM data were collected and analyzed using the Smart 3.0 animal behavior system (Panlab, Holliston, MA). The circular maze pool was divided into four and filled with clear water at 25 ± 2 °C. The swimming ability of mice was tested before the training phase, and a target platform was set up about 1 cm below the surface for the mice to stand on. In the training phase, each mouse underwent three trials daily for six consecutive days. In each trial, mice were given 60 s to find the platform. The time spent to reach the target platform (latency time) was recorded as one of the indicators of memory/learning indicator. On the seventh day, the platform was removed from the pool and each mouse was allowed to swim freely for 60 s. The time spent in the target platform quadrant was calculated as another indicator of spatial memory.

### 2.11. Immunohistochemistry and immunofluorescence

Immunohistochemistry and immunofluorescence experiments were performed as previously described (Wang et al., 2017). In brief, paraffin-embedded coronal brain sections (10 μm) from 4 to 5 animals in each group were prepared, with the operation site near the hippocampus being the main target sampling area. The sections were stained with hematoxylin and eosin to assess pathological changes in the brain, and with the Nissl stain to evaluate the changes in Nissl bodies. Samples were then observed using a light microscope (DM4B; Leica, Wetzlar, Germany). For immunofluorescence analysis, the sections were blocked with 5% fetal bovine serum and then incubated with the appropriate primary antibodies (dilution, 1:100) overnight at 4 °C. The next day, the sections were washed with PBST and then incubated with the appropriate secondary antibodies at room temperature for 2 h. Thereafter, the slices were co-incubated with DAPI solution for 5 min in order to stain the nuclei. Images were obtained using a fluorescence microscope. At least three images from four different animals in each group were analyzed.

### 2.12. Western blot

For *in vitro* experiments, proteins were extracted from SH-SY5Y cells incubated in six-well plates and grouped as described previously. For *in vivo* experiments, hippocampi of three to six mice from each group were homogenized in RIPA buffer containing 1 mM protease inhibitor cocktail and PMSF and centrifuged at 12,000 rpm for 30 min. Protein concentrations were measured using a BCA protein assay kit. Proteins were separated on 12% SDS polyacrylamide gels and transferred to polyvinylidene fluoride membranes. The membranes were blocked with 5% nonfat dry milk for 2 h, incubated with specific primary antibodies overnight at 4 °C, washed with TBST buffer, and incubated at room temperature for 1 h with a HRP-conjugated secondary antibody. Immunoreactive polypeptides were probed using enhanced chemiluminescence and the ChemiDoc MP Imaging system (734BR4330, Bio-Rad, Singapore). Finally, protein band intensities were quantified using the Image Lab software (6.0.0V, Bio-Rad, Hercules, CA).

### 2.13. Statistical analysis

Data are reported as the mean ± standard deviation of at least three independent experiments. Histograms and line graphs were analyzed using Prism 5 (GraphPad, San Diego, CA). For all data, differences were evaluated using one-way analysis of variance, and post hoc comparisons were performed using Bonferroni’s correction. Differences *p* values <.05 were considered statistically significant.

## 3. Results

### 3.1. GO and GO@Dau nanoparticles characterizations

**Figure 2.**
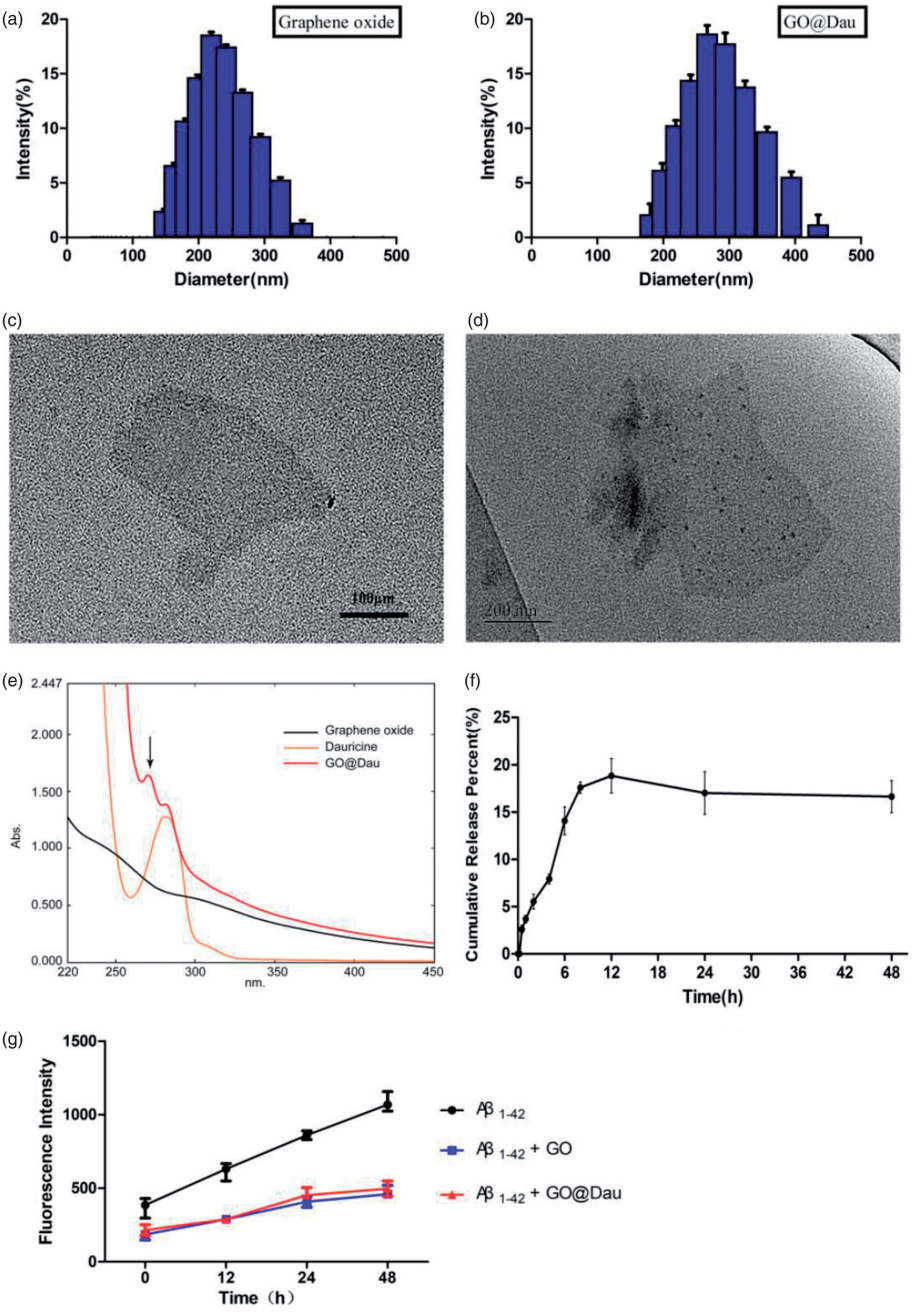
Characterization of graphene oxide (GO) and graphene oxide loading dauricine (GO@Dau). Particle size distribution of (a) GO and (b) GO@Dau nanoparticles by DLS. While transmission electron microscope images of (c) GO and (d) GO@Dau samples. (e) UV absorption spectrum of GO and GO@Dau. (f) Cumulative release of GO@Dau in PBS, pH = 7.4. (g) The fluorescence intensity of thioflavin (THT) binded β-amyloid (*n* = 3).

The standard techniques were used to characterize GO and GO@Dau nanoparticles in this study, including TEM, particle size distribution, zeta potential, drug loading percentage, and encapsulation efficiency percentage. Dynamic light scattering analysis confirmed that the average GO particle size was 158.76 ± 1.99 nm, which increased to 250.91 ± 15.16 nm after Dau loading (Figure 2(a,b)). Zeta potentials of GO and GO@Dau nanoparticles were −18.0 ± 0.44 mV and −19.8 ± 0.72 mV, respectively. Transmission electron microscopy images of GO and GO@Dau at 25 °C (Figure 2(c,d)) show that pristine GO had a flaky, paper-like texture, whereas almost no stacked sheets are observed. The appearance of GO@Dau was basically indistinguishable from that of GO. GO@Dau nanoparticles were successfully prepared with a percentage drug entrapment of 73.74 ± 4.91% and percentage drug loading of 6.98 ± 1.20%. The *in vitro* release properties of GO@Dau over 48 h were evaluated in a 0.1% Tween 80 PBS solution (pH 7.4) at 37 °C to mimic physiological conditions. The UV-visible absorption spectrum of GO@Dau showed the characteristic absorption peak of Dau at 298 nm (Figure 2(e)). As shown in Figure 2(f), rapid drug release was observed in the initial 12 h period, followed by sustained release over the next 12 h. At the same time, we carried thioflavin (THT) binding assay to detect the aggregation of β-amyloid peptide. Both GO and GO loaded on Dau effectively inhibited the abnormal aggregation and misfolding of amyloid (Figure 2(g)).

### 3.2. GO@Dau had neuroprotective effect on SH-SY5Y cell induced by Aβ_1-42_ treatment

**Figure 3.**
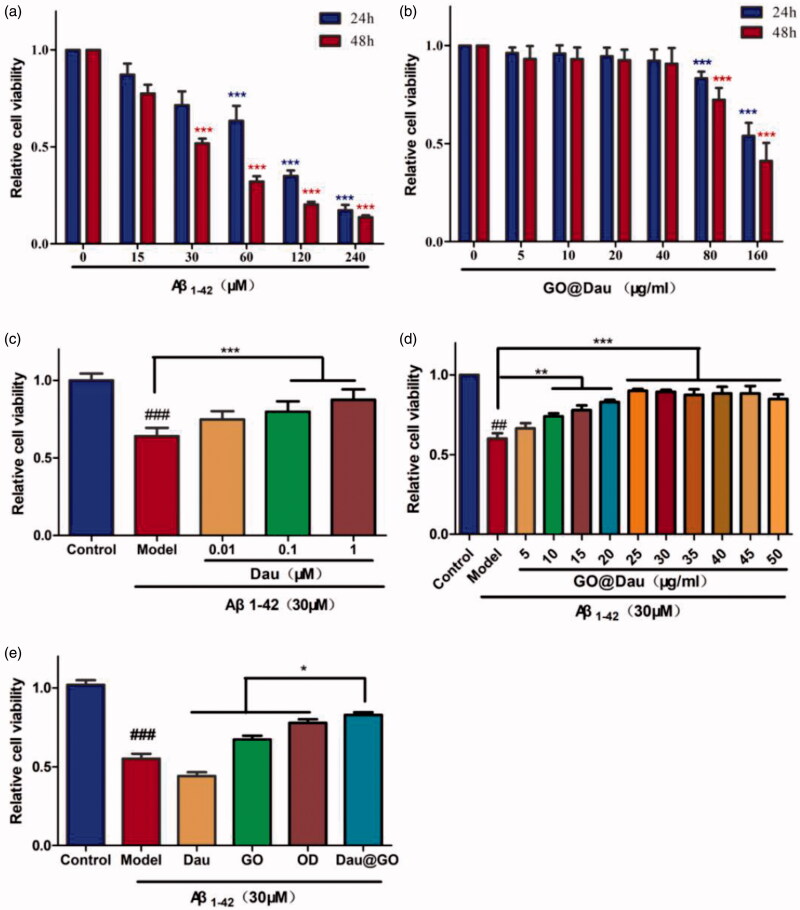
Effect of GO@Dau on neuroprotective in Aβ_1-42_-induced SH-SY5Y cell. (a) SH-SY5Y cells were treated with Aβ_1-42_ for 24 h and 48 h. (b) SH-SY5Y cells were treated GO@Dau with for 24 h and 48 h. (c) SH-SY5Y cells were treated with Aβ_1-42_ and incubated with dauricine for 48 h. (d) SH-SY5Y cells were treated with Aβ_1-42_ and incubated with GO@Dau for 48 h. (e) Aβ_1-42_-induced in SH-SY5Y cells treated with Dau (dauricine: 1.75 μg/mL), OD (dauricine: 1 μM), GO (23.25 μg/mL), and GO@Dau (25 μg/mL). (*n* = 6, ^###^*p* < .001, *p < .05, **p < .01, *p < .001).

Based on the anti-inflammatory and antioxidant effects of Dau and the ability of GO to inhibit amyloid aggregation, we hypothesized that GO@Dau would reverse the nerve cell apoptosis induced by Aβ_1-42_. First, the cytotoxicity of GO@Dau against SH-SY5Y cells was investigated using the CCK8 assay. As shown in Figure 3(a), no obvious toxicity was observed when the concentration of GO@Dau was lower than 60 μg/mL, confirming the safety threshold of the prepared nanocomposite. In the same way, we determined the induction concentration of Aβ_1-42_ (Figure 3(b)) and the optimal protective concentrations of Dau (Figure 3(c)) and GO@Dau against injured cells (Figure 3(d)). Finally, we incubated Dau, GO, and GO@Dau with Aβ-induced SH-SY5Y cells for 48 h. Here, we chose two concentrations of Dau, one is the optimal therapeutic concentration, and the other is the amount contained in GO@Dau. The results in Figure 3(e) show that GO@Dau can better rescue Aβ_1-42_-induced cell damage.

### 3.3. GO@Dau reduced intracellular ROS accumulation and reversed MDA and SOD level

**Figure 4.**
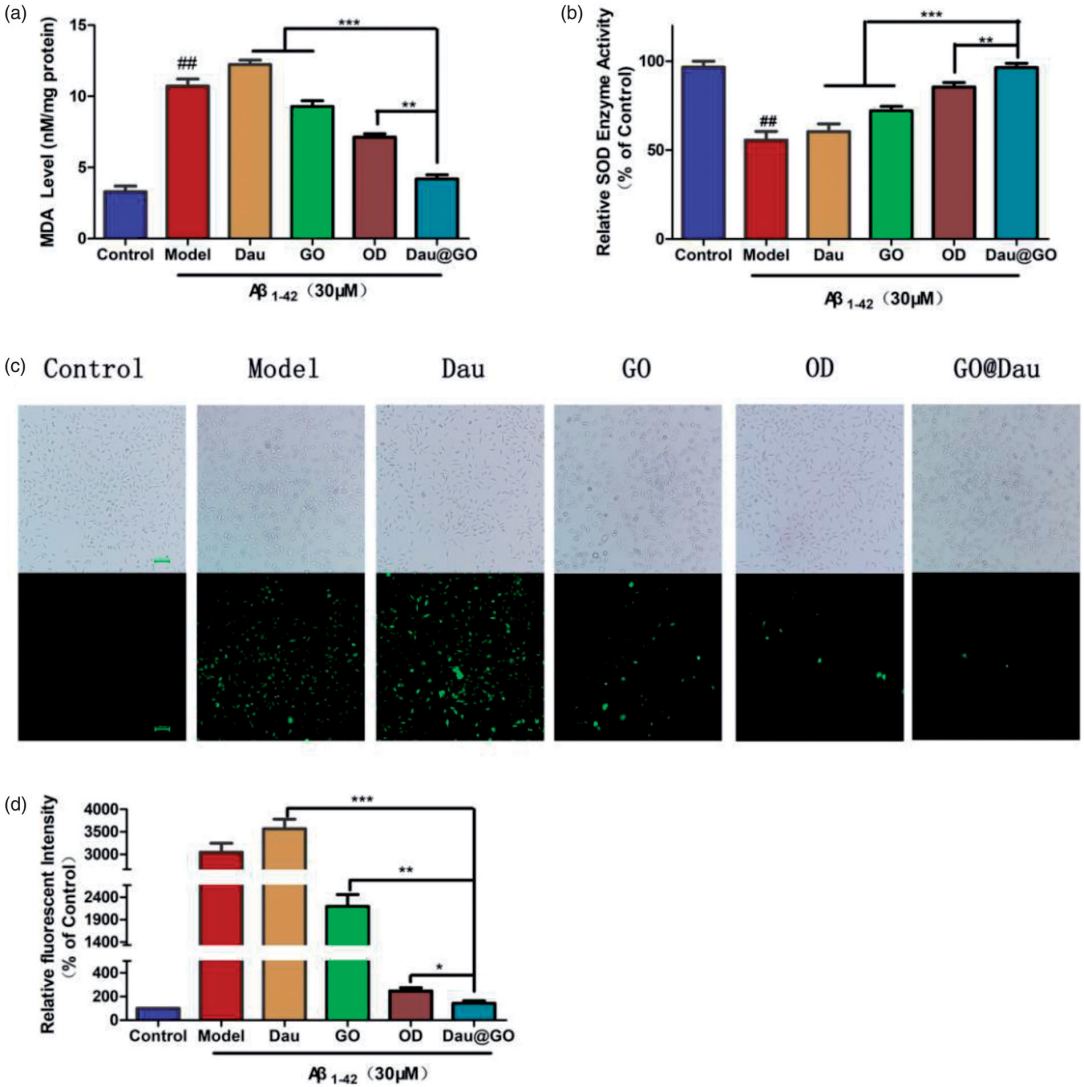
Effect of GO@Dau on Aβ_1-42_-induced oxidative stress in SH-SY5Y cells. (a) SOD activity and (b) MDA level in the SH-SY5Y cells were detected after treatments. (c) The fluorescence images of ROS generation, the green fluorescence represents ROS. (d) ROS responsiveness quantification (*n* = 3, *^##^p* < .01compared to control, *p＜.05,**p < .01 and ***p < .001).

The intracellular ROS levels were using DCFH-DA, which fluoresces when oxidized by ROS (Figure 4(a)). The ROS fluorescence of GO@Dau group was obvious lower than the other three treatment groups and had statistical significance. The relative level of MDA and SOD was measured by MDA and SOD detection kit, respectively. As shown in Figure 4, the SOD activity was reduced but the MDA level was elevated in the GO@Dau group when compared with the other treatment group (*p*<.05).

### 3.4. GO@Dau reduced apoptosis of SH-SY5Y cells

**Figure 5.**
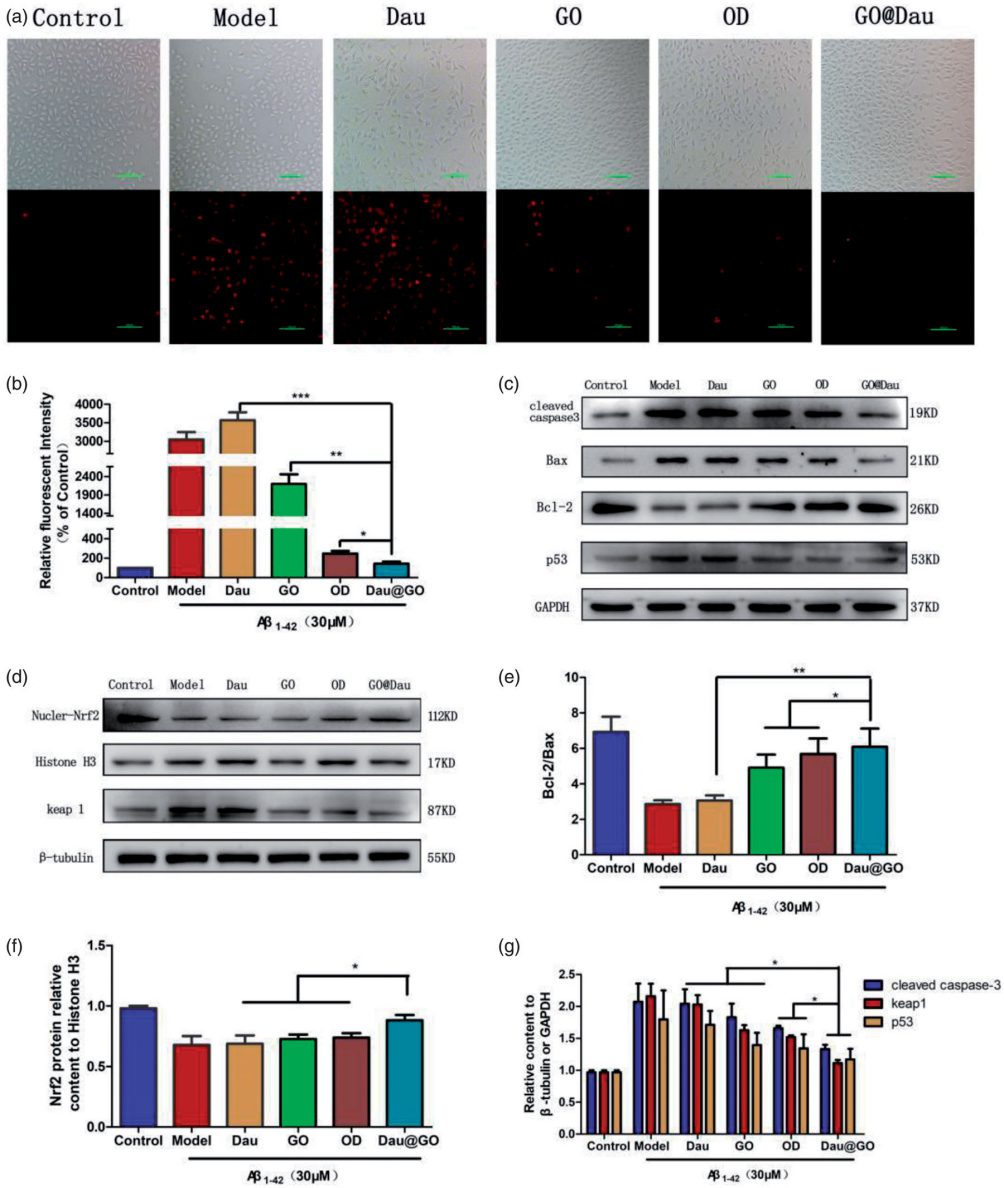
Effect of GO@Dau on Aβ_1-42_-induced apoptosis in SH-SY5Y cells. (a) The fluorescence images of TUNEL staining displayed apoptotic cells in different group, the red fluorescence represents positive apoptotic cells. (b) Quantitative of fluorescence. (c) Western blot analysis of Bcl-2, Bax, and cleaved caspase-3. (d) Western blot analysis of Nrf2 and Keap1. (e–g) Quantitative of blots (*n* = 3, *p< .05, **p< .01, and ***p< .001).

Apoptosis was assessed using TUNEL staining. As shown in Figure 5, there were almost no TUNEL-positive (apoptotic) cells in the control group, with an significant increase in TUNEL-positive cells in the model and Dau groups; moreover, the TUNEL-positive signal decreased in the latter three treatment groups, among which group GO@Dau is the most obvious. Next, we examined the expression of the pro-apoptosis proteins Bax and cleaved caspase-3 as well as the anti-apoptosis protein Bcl-2 using western blotting analysis (Figure 5(c)). Caspase-3 and p53 play important roles in the transduction of apoptosis signals. Bcl-2 inhibits apoptosis, while Bax enhances it; both of them are Bcl-2 family proteins and are important regulators of apoptosis. A decrease in the Bcl-2/Bax ratio is often used to judge the levels of apoptosis.

GO@Dau could significantly increase Bcl-2 expression and decreased Bax and caspase-3 expression in Aβ_1-42_-induced SH-SY5Y cells; the Bcl-2/Bax ratio was also increased (Figure 5(d,f)). We also examined the protein level of nuclear Nrf2 – its level in the GO@Dau group increased significantly from that in the control group (Figure 5(f)). Keap1 level was also decreased markedly in the GO@Dau group (Figure 5(g)).

### 3.5. Drug was delivered to the mice brain by intranasal administration

**Figure 6.**
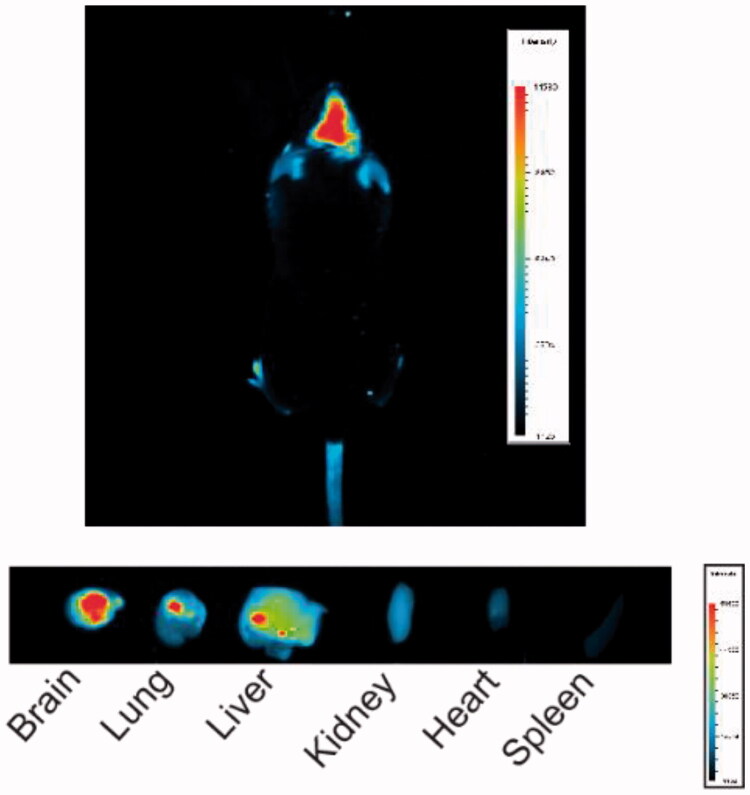
Representative *in vivo* imaging after intranasal administration.

*In vivo* imaging data from red fluorescent rhodamine B-loaded GO showed that it accumulated in the brain after intranasal administration (Figure 6(a)).

### 3.6. GO@Dau improved the anxiety-like behavior and neurocognitive deficits in aggregated Aβ_1-42_-induced mice

To investigate whether GO@ Dau could alleviate the anxiety-like behavior and neurocognitive deficits induced by aggregated Aβ_1-42_, OFT and MWM experiments were conducted. According to the results of the OFT, there was no difference in the initial total distance traveled among the groups initially (Figure 1(c)), indicating that mice from all groups had normal exercise activity and eliminating the possibility of any surgery-induced motor disturbances. However, compared with mice injected with saline into the hippocampus, mice with injected aggregated Aβ_1-42_ had significantly reduced movement in the central region (Figure 1(d)), whereas the administration of GO@Dau increased the cumulative time spent in the central region significantly as compared to that in the other administration groups. Thus, GO@Dau could better alleviate the anxiety-like behavior of AD mice.

In the MWM task, the swimming speed of the mice was first assessed, and the experimental data showed that there were no significant differences between the groups. Thereafter, we assessed the latency of the mice landing on the target platform during the training period, as well as the time spent in the target quadrant and the trajectories of the mice once the platform was removed after the training. The results showed that GO@Dau significantly alleviated the cognitive dysfunction induced by aggregated Aβ_1-42_.

### 3.7. GO@Dau ameliorated the damage of neurons in aggregated Aβ_1-42_-induced mice

**Figure 7.**
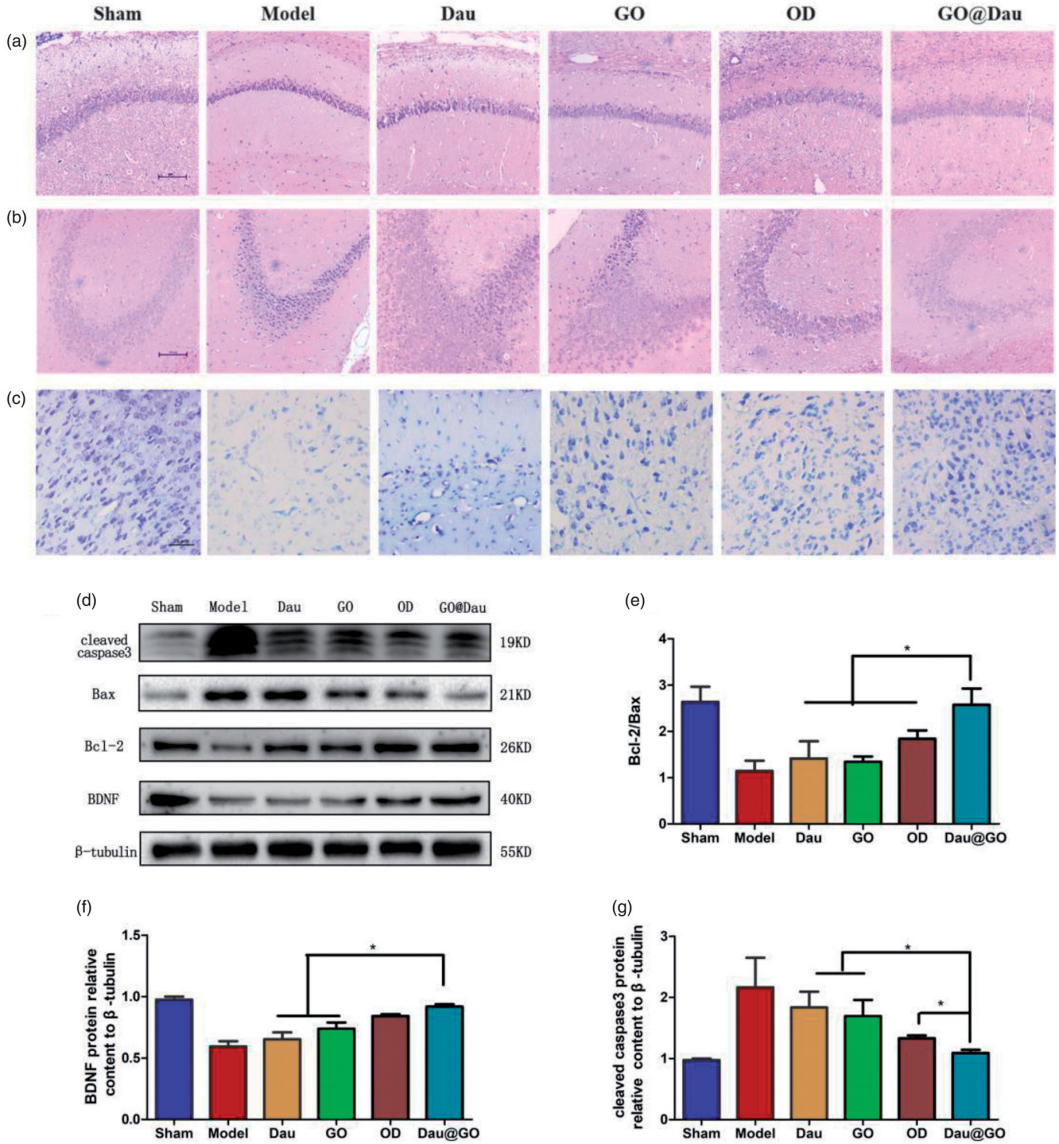
Effects of GO@Dau on neuronal loss in mice treated with aggregated Aβ_1-42_. Representative images of HE staining of the hippocampal (a) CA1 and (b) CA3 region were shown (scale bar = 100 μm). (c) Representative images of Nissl staining of the brain tissue (scale bar = 25 μm). (d) Western blot analysis of Bcl-2, Bax, cleaved caspase-3, and BDNF expression in brain tissue. (e, f) Quantitative of the blots (*n* = 3, *p< .05).

After the behavioral tests, we examined the neurons in the brain of the mice using hematoxylin eosin staining and Nissl body labeling, and observed that GO@Dau had certain neuroprotective effects. After induction with aggregated Aβ_1-42_, the neuronal nuclei were deeply stained (Figure 7(a)) and the number of cells (Figure 7(b)) and Nissl bodies was significantly reduced. However, GO@Dau reversed these pathological changes and had a better protective effect on the nerve cells than Dau or GO alone. We also determined the levels of cleaved caspase-3, Bax, Bcl-2, and BDNF, which are closely related to neuronal apoptosis, in the brain tissue of the mice. The results showed that GO@Dau could up-regulate the levels of Bcl-2 and BDNF and down-regulate the levels of cleaved caspase-3 and Bax (Figure 7(c)).

### 3.8. GO@Dau reduced the activation of microglia and astrocytes in aggregated Aβ_1-42_-induced mice

**Figure 8.**
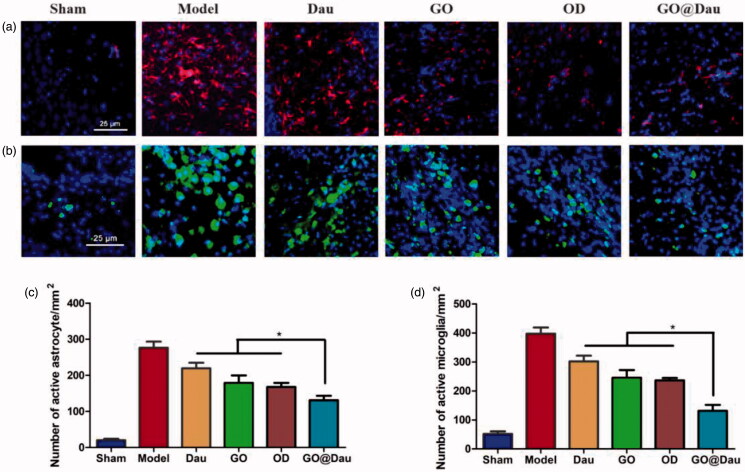
Ability of GO@Dau to reverse Aβ_1-42_-induced activation of microglia and astrocytes. (a) Immunofluorescence micrograph of GFAP expressing astrocytes. (b) Immunofluorescence micrograph of Iba-1 expressing microglia. The quantitative analysis of (c) GFAP and (d) Iba-1 immunofluorescence (*n* = 4 in each group, *p< .05).

Subsequently, we also investigated the activation of microglia and astrocytes in the brain to elucidate the mechanism by which GO@Dau reverses brain injury in aggregated Aβ_1-42_-induced mice. Under normal physiological conditions, microglia are in a resting state and their morphology is branched. When the central nervous system is damaged, microglia are activated and transform into mature immunocompetent cells that migrate to the injured site. Similarly, the morphology and biological functions of astrocytes also change, and benign ‘resting astrocytes’ turn into ‘reactive astrocytes’ in aggregated Aβ_1-42_-induced mice. GO@Dau led to significantly fewer activated microglia and astrocytes in aggregated Aβ_1-42_-induced mice than in mice in other treatment groups (Figure 8).

## 4. Discussion

The aim of this study was to verify the coordinated therapeutic effects of GO@Dau nanoparticles in AD models. Recent years, there are many natural product derived small molecules and polymer based multi-target therapeutic strategies (Rajasekhar et al., 2018, 2020; Samanta et al., 2019; Datta et al., 2020). As one of the main pathogenic mechanisms of AD, Aβ amyloid precipitation has always been the focus of AD treatment research (Rajasekhar et al., 2015; Rojo et al., 2017; Rajasekhar & Govindaraju, 2018). Several *in vivo* studies have reported the efficacy of anti-amyloid therapies; however, single anti-Aβ aggregation therapies are often ineffective owing to the complexity of the pathogenesis of AD (Chakraborty et al., 2016; Cui et al., 2017). Thus, it is necessary to develop novel drugs, administration strategies, and multi-target therapies.

One of the strategies in this study is drug repurposing, and many scholars have proposed that there is an inverse relationship between cancer and AD (Pandurangan et al., 2020; Moorthy & Govindaraju, 2021). Hence, we are committed to explore the anti-cancer drug Dau for its role in AD. In previous studies, our team found that Dau could regulate the Nrf2/Keap1 pathway to ameliorate the oxidative stress induced by Cu^2+^ ions or paraquat, and could also alleviate endoplasmic reticulum stress injury in a nematode model of AD by activating the IRE-1/XBP-1 and PERK/EIF2 pathways.

GO is a chemical derivative of graphite with unique physical and chemical properties, such as high stability and specific surface area and good biocompatibility. It has been widely used for bioimaging, biosensing, and other biological and medical applications (Sun et al., 2018; Abbas et al., 2019; Buskaran et al., 2020), one of which involves leveraging its various non-covalent interactions to load drugs for use as a potential drug carrier (Xia et al., 2019; Maciel et al., 2020). In early studies, it was discovered that the drug-resistance of breast cancer MCF-7/ADR cells for doxorubicin, a common antibiotic and antitumor drug, could be reversed through its π–π stacking and hydrogen bonding adsorption on the GO surface. Studies have also shown good anti-tumor therapeutic efficacies of the anti-HER-2 antibody and miR33a/miR199 adsorbed on the GO surface (Liu et al., 2018; Xiao et al., 2019).

The role of GO in the treatment of AD has mostly been limited to its use as a sensing material for the early diagnosis of mild cognitive impairment, although a few studies have investigated its direct pharmacological effects (Zhou et al., 2018). Yang et al. simulated and studied the mechanism by which GO inhibits the aggregation of Aβ_1-42_ and found that GO could penetrate into Aβ fibers and disrupt their structure, leading to their degradation. Subsequently, Li et al. also confirmed that GO could enhance the clearance of amyloid protein by inducing microglial and neuronal autophagy, indicating that GO may have the potential for direct application in AD therapies (Li et al., 2020).

Therefore, considering the biological properties of GO, the purpose of this study was to synthesize GO@Dau for AD therapy use. In this study, GO@Dau nanoparticles were prepared by co-incubating Dau and GO at 37 °C for 48 h. The drug loading rate was 6.98 ± 1.20%, and the cumulative release rate reached 13.01%, indicating that, due to the properties of GO, GO@Dau exhibited a sustained-release effect. We hypothesize that the methoxy-substituents on the phenyl ring of Dau form non-covalent bonds with GO for adsorption. However, the exact nature of the bonds between Dau and GO still needs to be determined. We had confirmed that GO@Dau inhibited the aggregation of the Aβ peptide using the ThT fluorescence test (He et al., 2019). In the Aβ_1-42_-induced cellular model in this study, it was found that, compared with Dau or GO alone, GO@Dau significantly decreased the levels of ROS and MDA in SH-SY5Y cells and significantly up-regulated the cell survival rate as well as the levels of SOD and Nrf2/Keap1 pathway-related proteins. *In vivo*, GO@Dau effectively improved the cognitive impairment and pathological damage in AD mice, up-regulated the expression of BDNF, and inhibited the activation of microglia and astrocytes induced by Aβ_1-42_. Based on these results, we believe that GO@Dau is a drug delivery system worthy of further development.

Interestingly, our study also yielded some other intriguing results. It is well known that intracerebral diseases are difficult to cure. Besides the complexities of the various pathogenic mechanisms, the specificity of the physiological structure of the human brain is also an important obstacle in the development of various therapeutic drugs. The blood–brain barrier is the protective barrier of the central nervous system; it is composed of vascular endothelial cells, pericytes, and glial cells and is mainly responsible for material exchange between the blood and brain tissues (Sarkar et al., 2017). The blood–brain barrier only allows hydrophobic molecules with a molecular mass less than 400 to pass; thus, more than 98% of small molecule drugs cannot pass through it, creating significant difficulties in the drug development process (Rohrer et al., 2018). In this study, we found that GO@Dau cannot penetrate the blood–brain barrier, so we attempted to administer it nasally (Serralheiro et al., 2014; Bahman et al., 2019; Espinoza et al., 2019). In order to prove that nasally administered GO@Dau can enter the brain, we added rhodamine B to GO as a fluorescent marker. *In vivo* imaging results showed that GO@RhB can be effectively enriched in the brain after nasal administration. Considering the characteristics of the nasal-brain transmission pathway, we believe that GO@Dau is internalized by olfactory neurons through endocytosis or pinocytosis into the olfactory bulb and then released and distributed to different regions of the brain. This will be verified in subsequent experiments (Chalansonnet et al., 2018; Tiozzo Fasiolo et al., 2019); however, there is no doubt that our results prove that GO-loaded drugs can enter the brain and function effectively after intranasal administration. In the future, it may also be possible for drugs like doxorubicin to be carried by GO into the brain in this manner for the treatment of gliomas or other central nervous system diseases.

**Scheme 1.**
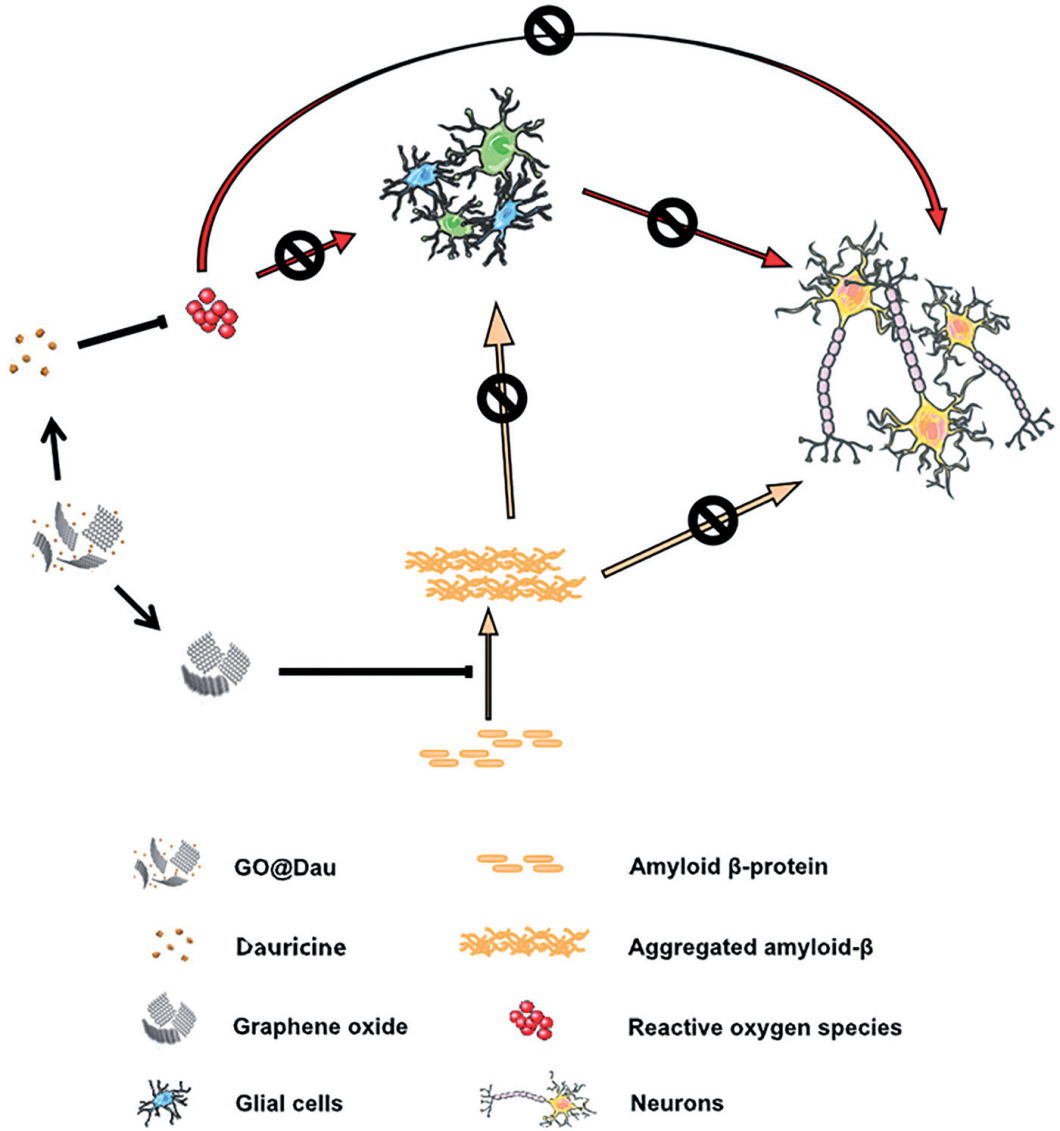
Schematic of the composition GO@Dau based on graphene oxide loaded with the neuroprotective agent dauricine. The graphene oxide inhibits abnormal accumulation of amyloid protein in brain, dauricine protects neurons against inflammation and antioxidation, both of them play a role in protecting AD from brain injury. The present study was carried out in model of AD.

In conclusion, here we describe the successful synthesis of GO@Dau and its curative effects in models of AD (Scheme 1). Both in the SH-SY5Y cell model and the Aβ_1-42_-induced AD mouse model, it was verified that GO-Dau regulated the Nrf2/Keap1 anti-oxidative pathway and had a better effect on AD compared with GO or Dau alone. At the same time, we also found that GO@Dau could enter the brain after nasal administration in AD mice. Modifying and optimizing the GO@Dau drug-delivery system would be of great significance for the development of more effective therapeutic strategies for AD.
